# Association between insulin resistance and prostate volume: A 4‐year analysis from the Reduction by Dutasteride of Prostate Cancer (REDUCE) Trial

**DOI:** 10.1002/bco2.70085

**Published:** 2025-09-14

**Authors:** James P. Daniels, Alexander Hernández‐Tirado, James Mirocha, Renning Zheng, Jordan Palmer, Daniel Moreira, Stephen J. Freedland

**Affiliations:** ^1^ Department of Urology University of Louisville Louisville Kentucky USA; ^2^ Department of Urology Cedars‐Sinai Medical Center Los Angeles CA USA; ^3^ Samuel Oschin Comprehensive Cancer Institute Cedars‐Sinai Medical Center California Los Angeles USA; ^4^ Clinical and Translational Science Institute Cedars‐Sinai Medical Center California Los Angeles USA; ^5^ School of Medicine, Tsinghua Medicine Tsinghua University Beijing China; ^6^ A.T. Still University College of Osteopathic Medicine Kirskville MO USA; ^7^ The University of Illinois College of Medicine Chicago Illinois USA; ^8^ Section of Urology Durham VA Medical Center Durham North Carolina USA

**Keywords:** Benign Prostatic Hyperplasia, HOMA‐IR, Insulin Resistance, Prostate Growth, Prostate Volume, REDUCE Trial

## Abstract

**Objectives:**

Most, but not all studies, suggest insulin resistance is associated with benign prostatic hyperplasia, but its impact on prostate volume (PV) changes over time remains unclear. We examined whether higher insulin resistance, measured by Homeostatic Model Assessment of Insulin Resistance (HOMA‐IR), is associated with larger PV and greater prostate growth over a 4‐year period.

**Materials and Methods:**

We analysed data from the 4‐year, randomized, double‐blind, placebo‐controlled REDUCE trial testing whether dutasteride could prevent prostate cancer. Patients underwent transrectal ultrasound measuring PV at baseline, year 2 and year 4. We calculated HOMA‐IR from baseline fasting glucose and insulin, then stratified patients into quartiles within each arm (placebo vs. dutasteride). Using multivariable models, we estimated PV changes over time. We conducted a sensitivity analysis excluding patients with diabetes.

**Results:**

Higher HOMA‐IR quartiles were associated with larger PV at baseline, year 2 and year 4 in both placebo and dutasteride arms (all p < 0.001), though absolute differences were modest. PV increased in the placebo arm over 4 years, whereas it decreased in the dutasteride arm. However, there was no significant association between HOMA‐IR and PV change in either arm. Results remained unchanged after excluding patients with diabetes.

**Conclusion:**

Patients with higher HOMA‐IR had modestly larger PVs at baseline, year 2 and year 4, but insulin resistance was unrelated to PV change over four years. These findings suggest that insulin resistance may be a modifiable risk factor contributing to benign prostatic enlargement, though further research is needed to determine its clinical relevance.

## 1 INTRODUCTION

Benign prostatic hyperplasia (BPH), the most common benign neoplasm in American patients with prostates and a common cause of lower urinary tract symptoms (LUTS), is a prevalent condition affecting millions worldwide, particularly patients over the age of 50.^1^ In the private healthcare sector alone, the economic burden of BPH and the often‐associated LUTS is estimated to be $4 billion annually in the US.^2^ Despite its prevalence and socioeconomic impact, the pathophysiology of BPH is only partly understood. While it is known that its prevalence increases with age, identifying other risk factors has proven challenging, with suggested risk factors including sedentary lifestyle, lack of exercise, smoking, excessive alcohol consumption, hypertension, type 2 diabetes, hyperlipidaemia and central obesity.^3^

Insulin resistance, a key component of metabolic syndrome and type 2 diabetes mellitus (T2DM), is defined as a decreased response to insulin that often requires higher insulin levels to achieve the integrated glucose‐lowering response. Beyond its role in glucose regulation, insulin has garnered attention for its impact on various physiological processes, such as cardiovascular disease, kidney disease and reproductive health.^4^ While BPH/LUTS likely results from many interrelated factors, evidence suggests insulin resistance may play a role.^5^, ^6^, ^7^, ^8^ However, a significant challenge in studying risk factors for BPH/LUTS lies in the fact that while BPH and LUTS are related, they are separate conditions. As such, when trying to understand the mechanistic drivers of urinary difficulties, it is crucial to separate out BPH (i.e. prostate enlargement) from LUTS, which can be caused by many reasons, though BPH is one of the most common.

Currently, the two leading hypotheses of how insulin resistance may contribute to LUTS are by insulin‐induced hyperplasia of prostate epithelial and stromal tissue (i.e. BPH) and increased prostate smooth muscle tone moderated by sympathetic nerve activity.^9^, ^10^ However, there is not yet a clear distinction as to which mechanism is the main contributor. Specifically, when focused on prostate size (i.e. BPH), there are mixed data on whether metabolic syndrome, a constellation of conditions often including insulin resistance, is linked to increased prostate size.^6^, ^8^, ^11^, ^12^ Hyperglycaemia, hyperinsulinaemia, diabetes status and insulin resistance have all been studied regarding their proposed associations with BPH/LUTS to mixed results.^9^ Several studies suggest a positive association between glucose homeostasis measures, particularly insulin resistance and prostate size.^5^, ^11^, ^13^, ^14^ However, another study reported no significant correlation.^6^ As such, the association between insulin resistance and PV remains unclear. Moreover, no study to date has examined the association between insulin resistance and prostate volume (PV) change over time using serial PV measurements. This gap is noteworthy, as serial prostate size measurements provide a dynamic and precise method of assessing prostate size and growth over time, rather than relying on a single measure at one point in time.

Importantly, while insulin resistance and glucose are intertwined, individuals can have normal glucose but still present insulin resistance. Given our underlying hypothesis that insulin drives BPH pathophysiology, we completed a post hoc analysis of the Reduction by Dutasteride of Prostate Cancer Events (REDUCE) Trial to assess the association between insulin resistance and both prostate size at baseline and PV growth over time. We hypothesize that over a 4‐year period of follow‐up in REDUCE, patients with higher insulin resistance would have larger prostates at baseline and an increased PV growth over time.

## 2 MATERIALS AND METHODS

### 2.1 Study Design and Participants

The study population consists of patients from the REDUCE trial. Methods and trial results were previously published.^15^ To summarize, REDUCE was a 4‐year, randomized, double‐blind, multicentre, placebo‐controlled study of prostate cancer risk reduction in patients with a pre‐study negative prostate biopsy and an elevated PSA. Eligible patients were 50–75 years with a baseline PSA of 2.5–10 ng/ml (if 50–60 years) or 3.0–10.0 ng/ml (if >60 years), a single negative prostate biopsy (6–12 cores) at least 6 months prior to enrolment, PVs ≤ 80 ml and an International Prostate Symptom Score <25 or <20 if taking alpha blockers. Patients were excluded if they had a history of prostate cancer, high‐grade intraepithelial neoplasia or atypical small acinar proliferation. Patients were randomized to receive either dutasteride 0.5 mg or placebo daily for 4 years. PV was measured by transrectal ultrasound (TRUS) during pre‐enrolment biopsy and at per‐protocol biopsies at year 2 and year 4 of the study. PV was calculated by multiplying the length of the three main axes (anteroposterior, cephalocaudal and transverse) by π/6. Baseline serum glucose and insulin with fasting status at the time of blood draw were measured during a 6‐month window either before or after the screening visit.

**FIGURE 1:**
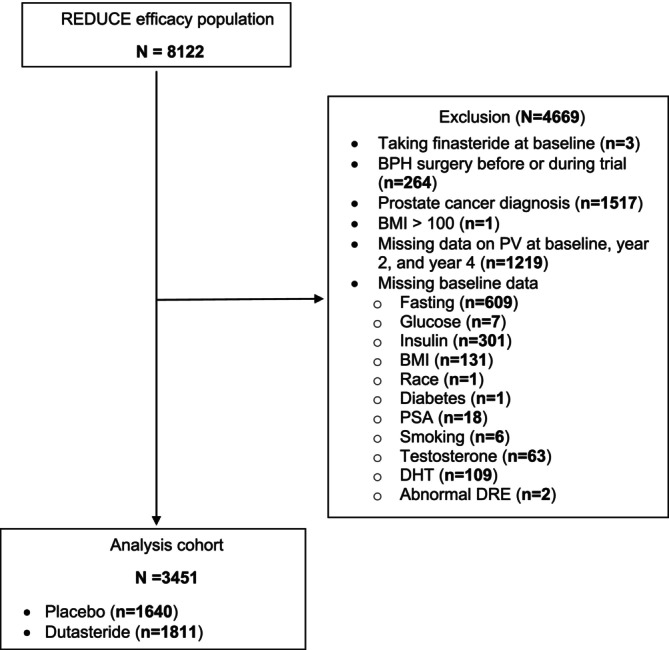
CONSORT Diagram Showing Participant Selection. Abbreviations: BMI = body mass index; BPH = benign prostatic hyperplasia; DHT = dihydrotestosterone; PSA = prostate‐specific antigen; PV = prostate volume; REDUCE = REduction by DUtasteride of prostate Cancer Events.

Further exclusions for our study include patients taking finasteride at baseline, diagnosed with prostate cancer during the REDUCE trial given the possible effect of prostate cancer on prostate size, history of BPH surgery prior to or during the study or missing baseline, year 2 or year 4 PV measurements. Patients missing baseline data on BMI, testosterone, dihydrotestosterone, race, diabetes history, smoking history, International Prostate Symptom Score or PSA were also excluded. Finally, patients with missing data on glucose, insulin, or who were not fasting at the baseline blood draw were excluded. Among 8122 patients in the efficacy population, 4669 were excluded, resulting in a final population of 3453 patients: 1641 in the placebo group and 1812 in the dutasteride group (Figure 1).

### 2.2 Statistical Analysis

HOMA‐IR for all included patients was calculated with the formula [fasting glucose (mmol/L) x fasting insulin (mU/L)/22.5]. Given the known effects of dutasteride on PV, patients were stratified into placebo and dutasteride (i.e. intervention) groups. Patients in both the placebo and dutasteride groups were then placed into quartiles dependent on their HOMA‐IR. Mean PV at baseline, year 2 and year 4 was analysed for patients in each quartile. We used the Cochran–Armitage trend test to assess linear trends for binary outcome variables reported across quartiles. We used contrasts to perform comparisons between groups for numerical outcome variables reported across quartiles. Since there is no universally accepted definition for what constitutes clinically significant prostate growth, we analysed PV growth as a continuous outcome. To assess change in volume over time, we estimated a multivariable longitudinal mixed model for PV growth from baseline to year 2 and baseline to year 4. The model adjusted for age at randomization (continuous), BMI at randomization (continuous), diabetes status (yes vs no), region of participants enrolled (Europe, North America and Other), race (White vs. Non‐White), smoking status (current, former and never), natural log of baseline PSA (continuous) and baseline IPSS (continuous). We performed a sensitivity analysis excluding subjects with reported or diagnosed diabetes given they often take medications that can affect glucose and insulin levels and hence HOMA‐IR; thus, the HOMA‐IR may not fully reflect the prostatic environment. Moreover, as the reason patients with DM were excluded in the sensitivity analysis was due to concerns specifically related to drugs that can alter insulin and/or glucose levels, we performed a second sensitivity analysis excluding patients taking any antidiabetic medications, whether they were documented as having DM or not. These medications included glibenclamide, tolbutamide, chlorpropamide, glipizide, gliclazide, glimepiride, gliquidone, metformin, phenformin, pioglitazone, rosiglitazone, repaglinide, nateglinide, acarbose, biguanides and insulin.

## 3 RESULTS

### 3.1 Characteristics of HOMA‐IR Quartiles

Higher HOMA‐IR quartiles were associated with larger baseline PV (p‐trend<0.0001), higher BMI (p‐trend<0.0001), diabetes (p‐trend<0.0001) and higher testosterone (p‐trend<0.0001) and DHT (p‐trend<0.0001). In contrast, higher HOMA‐IR was unrelated to age (p = 0.49), race (p = 0.22) and PSA (p = 0.67) (Table 1). When patients were stratified into placebo vs. dutasteride, overall similar trends were observed (Supplementary Tables S1 and S2).

**TABLE 1:** Baseline Characteristics of HOMA‐IR Quartile.

	1st Quartile (n = 874)	2nd Quartile (n = 852)	3rd Quartile (n = 864)	4th Quartile (n = 861)	p‐trend^1^
**HOMA‐IR**					NA
**Median**	1.3	2.2	3.2	5.9	
**IQR**	1.0, 1.5	1.9, 2.4	2.9, 3.5	4.8, 8.5	
**Baseline TRUS, ml**					<0.0001
**Mean (SD)**	43.6 (15.5)	45.4 (15.8)	46.2 (18.0)	48.3 (17.1)	
**IQR**	32.5, 53.3	33.7, 55.9	33.7, 56.9	35.4, 60.0	
**Age, years**					0.49
**Mean (SD)**	62.0 (6.0)	62.5 (6.0)	62.2 (5.9)	62.3 (6.1)	
**IQR**	57.0, 67.0	58.0, 67.0	57.0, 67.0	57.0, 67.0	
**BMI, kg/m** ^**2**^					<0.0001
**Median**	25.4	26.3	27.4	29.0	
**IQR**	23.5, 27.3	24.7, 28.4	25.5, 29.7	26.5, 31.7	
**Diabetes, no. (%)**	32 (3.7%)	47 (5.5%)	70 (8.1%)	150 (17.4%)	<0.0001
**Race, no. (%)**					0.22
**Other**	87 (10%)	62 (7%)	61 (7%)	72 (8%)	
**White**	787 (90%)	790 (93%)	803 (93%)	789 (92%)	
**PSA, ng/ml**					0.67
**Mean (SD)**	5.8 (1.9)	5.9 (1.9)	5.9 (1.9)	5.8 (1.9)	
**IQR**	4.2, 7.2	4.3, 7.3	4.3, 7.3	4.3, 7.2	
**Testosterone, nmol/L**					<0.0001
**Median**	16.6	15.2	13.6	12.8	
**IQR**	12.7, 21.2	11.7, 19.6	10.6, 18.2	9.9, 16.9	
**DHT, nmol/L**					<0.0001
**Median**	1.4	1.3	1.1	1.0	
**IQR**	1.0, 1.9	0.9, 1.8	0.8, 1.6	0.7, 1.5	

^1^ Contrast analysis for numerical variables, Cochran‐Armitage trend test for categorical variables.

### 3.2 Prostate Volume Through 4 Years

**FIGURE 2:**
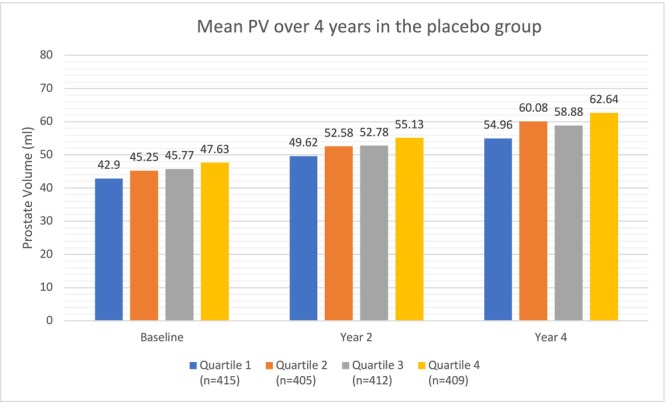
Mean PV over 4 years in the placebo group.

In the placebo group, higher HOMA‐IR quartiles were associated with larger PV at baseline (p‐trend<0.00001), year 2 (p‐trend = 0.0004) and year 4 (p‐trend<0.00001) (Figure 2, Table 2). However, the differences in PV between the highest and lowest quartiles were modest at baseline (47.63 ml vs 42.90 ml), year 2 (55.13 ml vs 49.62 ml) and year 4 (62.64 ml vs 54.96 ml).

**TABLE 2:** Mean prostate volume over 4 years in the placebo and dutasteride groups.

Treatment Arm	Prostate Volume	1st Quartile (n = 415)	2nd Quartile (n = 405)	3rd Quartile (n = 412)	4th Quartile (n = 408)	p‐trend^1^
**Placebo** **Group**	Baseline	42.90 ml	45.25 ml	45.77 ml	47.63 ml	<0.0001
	Year 2	49.62 ml	52.58 ml	52.78 ml	55.13 ml	0.0004
	Year 4	54.96 ml	60.08 ml	58.88 ml	62.64 ml	<0.0001
**Dutasteride** **Group**	Baseline	44.25 ml	45.47 ml	46.68 ml	48.88 ml	<0.0001
	Year 2	37.95 ml	38.78 ml	39.40 ml	41.84 ml	0.0005
	Year 4	37.85 ml	38.99 ml	39.56 ml	42.25 ml	0.0002

^1^ Contrast analysis.

**FIGURE 3:**
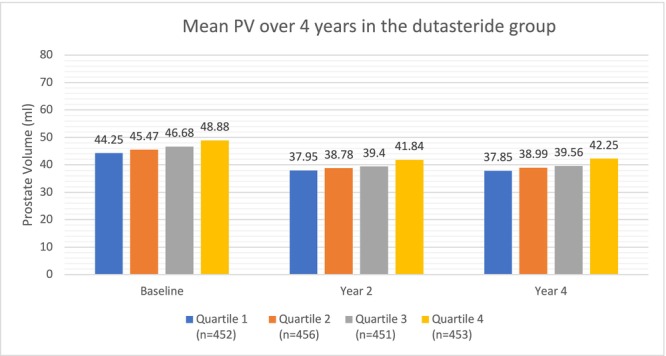
Mean PV over 4 years in the dutasteride group.

Similar to the placebo group, in the dutasteride group, higher HOMA‐IR quartiles were again associated with larger PV at baseline (p‐trend<0.0001), year 2 (p‐trend = 0.0005) and year 4 (p‐trend = 0.0002) (Figure 3, Table 2). However, similar to the placebo group, overall differences between the highest and lowest HOMA‐IR quartiles were modest. Of note, the differences in PV between the highest and lowest quartiles were slightly smaller than the placebo group at year 2 (41.84 ml vs 37.95 ml) and year 4 (42.25 ml vs 37.85 ml), most likely due to the effects of dutasteride.

### 3.3 Change in Prostate Volume at Year 2 and Year 4

In the placebo group, while PV increased from baseline to year 2 and from baseline to year 4 in all HOMA‐IR groups, there were no significant associations between higher HOMA‐IR quartiles and greater PV growth from baseline to year 2 (p = 0.99) and from baseline to year 4 (p = 0.11) (Table 3).

**TABLE 3:** Adjusted estimate of prostate volume change in the placebo and dutasteride groups.

Treatment Arm	HOMA‐IR	Year 2 PV change relative to baseline	95 %CI	Year 4 PV change relative to baseline (ml)	95% CI
**Placebo** **Group**	1st Quartile 2nd Quartile 3rd Quartile 4th Quartile Linear Contrast	6.71 ml 7.33 ml 7.01 ml 7.50 ml 0.99	5.25–8.17 5.69–8.97 5.54–8.48 5.88–9.12	12.06 ml 14.84 ml 13.11 ml 15.02 ml 0.11	10.48–13.63 12.88–16.79 11.28–14.94 12.98–17.05
**Dutasteride** **Group**	1st Quartile 2nd Quartile 3rd Quartile 4th Quartile Linear Contrast	−6.30 ml −6.69 ml −7.28 ml −7.04 ml 0.39	−7.40, −5.19 −7.76, −5.62 −8.55, −6.00 −8.41, −5.67	−6.39 ml −6.49 ml −7.12 ml −6.63 ml 0.64	−7.76, −5.03 −7.73, −5.24 −8.74, −5.49 −8.18, −5.09

Adjusted for baseline data on BMI, PSA, age, testosterone, DHT, smoking status, diabetes status, race, region, abnormal DRE and year.

In the dutasteride group, given the known effects of this drug, it is unsurprising that PV decreased from baseline to year 2 and from baseline to year 4 in all HOMA‐IR groups. However, similar to the placebo group, there were no associations between higher HOMA‐IR quartiles and PV change from baseline to year 2 (p = 0.39) and from baseline to year 4 (p = 0.64) (Table 3).

### 3.4 Sensitivity Analyses

Given diabetes can lead to altered HOMA‐IR measurements, we performed a sensitivity analysis to exclude subjects with diabetes. When this was done, overall associations remained largely unchanged. Higher HOMA‐IR quartiles were again associated with larger baseline, year 2 and year 4 PV in both the placebo and dutasteride groups (all p ≤ 0.001) (Supplementary Table S3, Supplementary Figure S1 and S2). There were no associations between higher HOMA‐IR quartiles and PV change from baseline to year 2 (all p ≥ 0.09) and from baseline to year 4 (all p ≥ 0.34) in either the placebo or dutasteride groups (Supplementary Table S4).

Additionally, given that antidiabetic therapy can alter both HOMA‐IR measurements and PV, we performed a sensitivity analysis to exclude subjects taking these medications. Overall associations remained unchanged, with higher HOMA‐IR quartiles associated with larger baseline, year 2 and year 4 PV in the placebo group (all p ≤ 0.001) (Supplementary Table S5). There were no associations between higher HOMA‐IR quartiles and PV change from baseline to year 2 (p = 0.80) and from baseline to year 4 (p = 0.43) in the placebo group (Supplementary Table S6).

## 4 DISCUSSION

Treatment for BPH and LUTS costs the private healthcare sector in the US up to $4 billion annually.^2^ Understanding the extent to which modifiable risk factors such as insulin resistance, a key component of the metabolic syndrome, contribute to BPH/LUTS could have dramatic public health implications. To better understand the role of insulin resistance in prostate growth, we performed a post‐hoc analysis of REDUCE. We found that subjects with higher insulin resistance had larger PV at baseline, year 2 and year 4 of the 4‐year study period in REDUCE. However, change in PV from baseline to year 2 or from baseline to year 4 was not related to insulin resistance. These data suggest insulin resistance may be a modifiable risk factor for prostate enlargement. The degree to which the larger prostates correlate with clinical symptoms (i.e. LUTS) requires further study.

We found that higher insulin resistance was associated with larger prostate size in REDUCE at baseline, year 2 and year 4. We performed two sensitivity analyses, one excluding subjects with diabetes and a second excluding subjects taking antidiabetic therapy because these medications may alter insulin resistance, fasting glucose and PV, thereby affecting HOMA‐IR and PV measurements and potentially confounding their association. After removing these patients, we noted that higher insulin resistance remained associated with larger PVs at baseline, year 2 and year 4, indicating that the association is not driven by diagnosed diabetes or its treatments. While there are conflicting data, our results align with the majority of the literature showing an association between insulin resistance and larger PV. A 2006 case–control study, which excluded diabetic and obese patients, found significantly higher HOMA levels in symptomatic BPH patients undergoing prostatectomy compared to healthy controls.^13^ Additionally, the study identified a modest yet significant correlation between HOMA and larger PV among BPH patients. Similarly, a 2020 study found that HOMA‐IR was significantly correlated with larger PV, but again, associations were modest.^11^ Likewise, the differences in PV across HOMA‐IR quartiles in our study, while statistically significant, were also modest, ranging from 43.6 ml in the lowest quartile to 48.3 in the highest HOMA‐IR quartile for baseline PV. As such, it is intriguing to note that the one study which did not find an association between HOMA‐IR and PV was among 369 patients.^6^ Thus, perhaps it was underpowered to detect modest associations. Alternatively, that study was comprised of only Black men, and thus whether HOMA‐IR correlates with PV in Black men requires further study.

Other studies found that metabolic syndrome, a complex of multiple conditions that often includes insulin resistance at its core, was also associated with prostate enlargement. A Swedish study that defined metabolic syndrome with obesity, type 2 diabetes, HDL‐cholesterol, treated hypertension and fasting insulin saw that patients with metabolic syndrome had greater PV than patients without metabolic syndrome (49.0 ml vs 28.5 ml).^16^ Moreover, compared to men presenting with none of the metabolic syndrome components, men with one or more metabolic syndrome components had larger PVs. Similarly, a more recent Korean cross‐sectional study of 968 men found that patients with abnormal fasting glucose (≥100 mg/dl or on hyperglycaemic treatment) and abnormal waist circumference (≥90 cm) had a larger PV compared to subjects with normal parameters, while other components of metabolic syndrome (hypertension, dyslipidaemia, hypertriglyceridemia) were not significantly related to PV.^17^ Collectively, the literature suggests there is an association between metabolic syndrome and PV and supports the potential impact of metabolic health on prostate health.

Further stratification by treatment arm, either placebo or dutasteride, yielded similar trends, with higher HOMA‐IR consistently associated with larger PV at baseline, year 2 and year 4. However, PV growth over time differed between arms. All HOMA‐IR quartiles in the placebo group showed increased PV at year 2 and year 4, compared to baseline. On the other hand, we observed an expected decreased in PV in all HOMA‐IR quartiles in the dutasteride group at year 2 and year 4, compared to baseline. Notably, the effect of dutasteride on PV reduction was consistent across all HOMA‐IR quartiles, indicating that it remains effective regardless of insulin resistance.

If causal, there are several potential mechanisms to explain the link between insulin resistance/hyperinsulinaemia and prostate enlargement. Specifically, alterations in multiple metabolic pathways have been suggested to play a role including increased sympathetic nervous system activation, elevated insulin‐like growth factor‐1 (IGF‐1) and altered steroidal hormones metabolism.^18^ Insulin promotes elevated plasma catecholamines, specifically norepinephrine levels, which can induce proliferation of non‐epithelial prostatic cells through the activation of pro‐proliferative signals. Insulin resistance and the compensating hyperinsulinaemia cause elevated plasma IGF‐1 that can also stimulate prostatic growth. Additionally, insulin can increase the transcription of androgen receptor target genes and enhance the expression of 5α reductase enzyme, boosting conversion of testosterone to DHT, a key prostate growth factor.

In our study, despite higher HOMA‐IR being associated with larger prostate size at baseline, HOMA‐IR was not significantly associated with the rate of prostate growth over a 2‐year or 4‐year time frame. Although the Swedish study previously discussed reported an increased growth rate over time,^16^ our findings are not inconsistent with these results. Specifically, they assumed everyone had a PV of 20 ml at 20 years of age and assessed annual prostate growth over the lifetime of the patient. In contrast, our study used actual PV measurements over a 4‐year time frame. As such, whether higher HOMA‐IR is associated with greater PV growth over the long term among older adults requires further study.

This study has some limitations. The study did not assess LUTS. Therefore, the clinical significance of the observed modest associations between insulin resistance and larger prostate size regarding LUTS requires further study. Additionally, our study population was predominantly White, which may limit the generalizability of our findings to other racial and ethnic groups. Study participants were selected based on the eligibility criteria of the REDUCE trial, which may introduce selection bias. Similarly, enrolment criteria capped PV to a maximum of 80 ml, limiting our ability to assess significantly larger prostates. Notably, prior studies that did not restrict PV at enrolment observed similar associations between higher fasting insulin or insulin resistance and larger prostate size.^11^, ^19^ Furthermore, this analysis focused on a 4‐year follow‐up period, which may not capture longer‐term associations between insulin resistance and prostate growth. Moreover, HbA1c data were not available, limiting our ability to assess chronic glycaemic control or severity of diabetes. Our study was also intentionally focused on insulin resistance (HOMA‐IR) rather than pancreatic β‐cell function (HOMA2‐B); while this choice directly addressed our primary hypothesis, it limits a more comprehensive assessment of the interplay between pancreatic function, insulin and PV. Likewise, although it would be ideal to exclude patients who developed diabetes during the study in the sensitivity analysis, diabetes diagnoses after baseline were unavailable. Also, IR is often viewed as part of the metabolic syndrome, which also includes low HDL, obesity and hypertension. While some of these factors have been examined in REDUCE previously as related to prostate volume/LUTS,^20^, ^21^ how these various factors interact to influence PV requires further study. Finally, we calculated HOMA‐IR using baseline measurements of insulin and glucose, which may not accurately reflect insulin resistance over the entire 4‐year study period.

## AUTHOR CONTRIBUTIONS

**James P. Daniels:** Concept; writing; intepretation. **Alexander Hernández‐Tirado:** Concept; writing; intepretation. **James Mirocha:** Statistical analysis. **Renning Zheng:** Statistical analysis. **Jordan Palmer:** Writing. **Daniel Moreira:** Interpretation and writing. **Stephen J. Freedland:** Concept; writing; intepretation; supervision.

## 5 CONCLUSION

This study investigated the association between insulin resistance, as measured by fasting HOMA‐IR, and PV in a large cohort of patients from the REDUCE trial. We found a significant but modest association between greater insulin resistance and larger PV in both the placebo and dutasteride groups at baseline, 2 years and 4 years follow‐up. However, HOMA‐IR was not associated with PV change over the 4‐year study period. While these results support the notion that insulin resistance may be a modifiable risk factor contributing to benign prostatic enlargement, further research is needed to determine its clinical relevance given the modest associations.

## CONFLICT OF INTEREST STATEMENT

None.

## Supporting information


**Table S1.** Baseline Characteristics of Placebo Group by HOMA‐IR Quartile.
**Table S2.** Baseline Characteristics of Dutasteride Group by HOMA‐IR Quartile.
**Table S3.** Mean Prostate Volume over 4 years in the placebo and dutasteride groups (excluding subjects with diabetes).
**Table S4.** Adjusted estimate of prostate volume change in the placebo and dutasteride groups (excluding subjects with diabetes).
**Table S5.** Mean Prostate Volume over 4 years in the placebo group (excluding subjects on medicines that might affect glucose/insulin levels).
**Table S6.** Adjusted estimate of prostate volume growth in the placebo group (excluding subjects on medicines that might affect glucose/insulin levels).
**Figure S1.** Mean PV over 4 years in the placebo group (excluding subjects with diabetes).
**Figure S2.** Mean PV over 4 years in the dutasteride group (excluding subjects with diabetes).
