# Supplementary material for: Association between insulin resistance and prostate volume: A 4‐year analysis from the Reduction by Dutasteride of Prostate Cancer (REDUCE) Trial
Source: BJUI Compass. 2025 Sep 14;6(9):e70085. doi: 10.1002/bco2.70085 (PMC12433711; doi:10.1002/bco2.70085)
Supplement: Supplementary file 1 — Table S1. Baseline Characteristics of Placebo Group by HOMA‐IR Quartile. Table S2. Baseline Characteristics of Dutasteride Group by HOMA‐IR Quartile. Table S3. Mean Prostate Volume over 4 years in the placebo and dutasteride groups (excluding subjects with diabetes). Table S4. Adjusted estimate of prostate volume change in the placebo and dutasteride groups (excluding subjects with diabetes). Table S5. Mean Prostate Volume over 4 years in the placebo group (excluding subjects on medicines that might affect glucose/insulin levels). Table S6. Adjusted estimate of prostate volume growth in the placebo group (excluding subjects on medicines that might affect glucose/insulin levels). Figure S1. Mean PV over 4 years in the placebo group (excluding subjects with diabetes). Figure S2. Mean PV over 4 years in the dutasteride group (excluding subjects with diabetes). [file BCO2-6-e70085-s001.docx]

| Supplementary Table 1. Baseline Characteristics of Placebo Group by HOMA-IR Quartile | | | | | |
| --- | --- | --- | --- | --- | --- |
|  | **1^st^ Quartile** (n=415) | **2^nd^ Quartile**  (n=405) | **3^rd^ Quartile** (n=412) | **4^th^ Quartile** (n=408) | **p-trend^1^** |
| HOMA-IR  Median  IQR | 1.2  1.0, 1.4 | 2.2  1.9, 2.4 | 3.3  3.0, 3.7 | 6.1  5.0, 9.0 | NA |
| Baseline TRUS, ml  Mean (SD)  IQR | 42.9 (15.4)  31.4, 53.0 | 45.3 (15.2)  33.9, 55.1 | 45.8 (15.5)  33.7, 56.4 | 47.6 (15.8)  35.3, 58.3 | <0.0001 |
| Age, years  Mean (SD)  IQR | 62.0 (5.8)  58.0, 68.0 | 62.1 (5.9)  58.0, 66.0 | 62.4 (6.0)  58.0, 67.0 | 62.2 (6.1)  57.0, 67.0 | 0.47 |
| BMI, kg/m^2^  Median  IQR | 25.4  23.6, 27.3 | 26.4  24.7, 28.5 | 27.3  25.4, 29.8 | 28.9  26.6, 31.6 | <0.0001 |
| Diabetes, no. (%) | 19 (4.6%) | 24 (5.9%) | 36 (8.7%) | 76 (18.6%) | <0.0001 |
| Race, no. (%)  Other  White | 36 (9%)  379 (91%) | 33 (8%)  372 (92%) | 36 (9%)  376 (91%) | 36 (9%)  372 (91%) | 0.87 |
| PSA, ng/ml  Mean (SD)  IQR | 5.8 (1.9)  4.2, 7.4 | 5.9 (2.0)  4.3, 7.4 | 5.9 (2.0)  4.3, 7.3 | 5.7 (1.9)  4.3, 6.9 | 0.42 |
| Testosterone, nmol/L  Median  IQR | 16.8  12.8, 21.5 | 15.1  11.5, 19.4 | 13.4  10.6, 18.2 | 13.2  10.2, 16.9 | <0.0001 |
| DHT, nmol/L  Median  IQR | 1.4  1.0, 1.9 | 1.3  0.9, 1.7 | 1.1  0.8, 1.6 | 1.0  0.7, 1.5 | <0.0001 |
| ^1^Contrast analysis for numerical variables, Cochran-Armitage trend test for categorical variables | | | | | |

| Supplementary Table 2. Baseline Characteristics of Dutasteride Group by HOMA-IR Quartile | | | | | |
| --- | --- | --- | --- | --- | --- |
|  | **1^st^ Quartile** (n=452) | **2^nd^ Quartile**  (n=456) | **3^rd^ Quartile** (n=450) | **4^th^ Quartile** (n=453) | **p-trend^1^** |
| HOMA-IR  Median  IQR | 1.3  1.0, 1.5 | 2.1  1.9, 2.4 | 3.1  2.9, 3.5 | 5.6  4.6, 8.3 | NA |
| TRUS, ml  Mean (SD)  IQR | 44.3 (15.6)  33.1, 53.7 | 45.5 (16.3)  33.2, 56.6 | 46.7 (20.1)  33.0, 57.6 | 48.9 (18.1)  35.6, 60.9 | <0.0001 |
| Age, years  Mean (SD)  IQR | 61.9 (6.1)  57, 67 | 62.9 (6.0)  59, 68 | 62.0 (5.9)  57, 67 | 62.3 (6.1)  57, 67 | 0.93 |
| BMI, kg,m^2^  Median  IQR | 25.3  23.5, 27.4 | 26.2  24.8, 28.0 | 27.4  25.5, 29.4 | 29.0  26.5, 31.8 | <0.0001 |
| Diabetes, no. (%) | 13 (2.9%) | 24 (5.3%) | 30 (6.7%) | 77 (17.0%) | <0.0001 |
| Race, no. (%)  Other  White | 51 (11%)  401 (89%) | 29 (6%)  427 (94%) | 24 (5%)  426 (95%) | 36 (8%)  417 (92%) | 0.06 |
| PSA, ng/ml  Mean (SD)  IQR | 5.8 (1.9)  4.2, 7.2 | 5.8 (1.9)  4.4, 7.0 | 5.8 (1.9)  4.2, 7.2 | 6.0 (1.9)  4.3, 7.4 | 0.25 |
| Testosterone, nmol/L  Median  IQR | 16.5  12.6, 20.9 | 15.4  12.1, 20.0 | 13.8  10.5, 18.3 | 12.6  9.8, 17.0 | <0.0001 |
| DHT, nmol/L  Median  IQR | 1.4  1.1, 1.9 | 1.3  0.9, 1.8 | 1.1  0.8, 1.6 | 1.0  0.7, 1.5 | <0.0001 |
| ^1^Contrast analysis for numerical variables, Cochran-Armitage trend test for categorical variables | | | | | |

| Supplementary Table 3. Mean Prostate Volume over 4 years in the placebo and dutasteride groups (excluding subjects with diabetes) | | | | | | |
| --- | --- | --- | --- | --- | --- | --- |
| Treatment Arm | Prostate Volume | **1^st^ Quartile** (n=366) | **2^nd^ Quartile** (n=378) | **3^rd^ Quartile** (n=369) | **4^th^ Quartile** (n=372) | **p-trend^1^** |
| Placebo group | Baseline | 43.17 ml | 45.54 ml | 45.31 ml | 47.97 ml | <0.0001 |
|  | Year 2 | 49.97 ml | 52.12 ml | 51.88ml | 55.68 ml | 0.0006 |
|  | Year 4 | 55.25 ml | 59.72 ml | 57.75 ml | 63.67 ml | <0.0001 |
| Dutasteride Group | Baseline | 44.04 ml | 45.52 ml | 46.69 ml | 48.04 ml | 0.0007 |
|  | Year 2 | 37.81 ml | 38.74 ml | 39.15 ml | 41.23 ml | 0.004 |
|  | Year 4 | 37.75 ml | 38.87 ml | 39.37 ml | 41.78 ml | 0.001 |
| ^1^Contrast analysis | | | | | | |

| Supplementary Table 4. Adjusted estimate of prostate volume change in the placebo and dutasteride groups (excluding subjects with diabetes) | | | | | |
| --- | --- | --- | --- | --- | --- |
| Treatment Arm | HOMA-IR | **Year 2**  **PV change relative to baseline** | **95 %CI** | **Year 4**  **PV change relative to baseline (ml)** | **95% CI** |
| Placebo Group | 1^st^ Quartile  2^nd^ Quartile  3^rd^ Quartile  4^th^ Quartile  Linear Contrast | 6.80 ml  6.58 ml  6.57 ml  7.71 ml  P=0.09 | 5.23-8.37  4.96-8.21  5.02-8.12  6.03-9.39 | 12.08 ml  14.18 ml  12.43 ml  15.70 ml  P=0.93 | 10.41-13.74  12.23-16.12  10.51-14.35  13.52-17.87 |
| Dutasteride Group | 1^st^ Quartile  2^nd^ Quartile  3^rd^ Quartile  4^th^ Quartile  Linear Contrast | -6.23 ml  -6.79 ml  -7.53 ml  -6.81 ml  P=0.24 | -7.38, -5.08  -7.83, -5.74  -8.92, -6.14  -8.26, -5.35 | -6.28 ml  -6.65 ml  -7.32 ml  -6.26 ml  P=0.34 | -7.73, -4.84  -7.83, -5.47  -9.11, -5.53  -7.83, -4.69 |
| Adjusted for baseline data on BMI, PSA, Age, Testosterone, DHT, smoking status, race, region, abnormal DRE and year | | | | | |

| Supplementary Table 5. Mean Prostate Volume over 4 years in the placebo group (excluding subjects on medicines that might affect glucose/insulin levels) | | | | | |
| --- | --- | --- | --- | --- | --- |
| Prostate Volume | **1^st^ Quartile**  (n=403) | **2^nd^ Quartile**  (n=374) | **3^rd^ Quartile**  (n=361) | **4^th^ Quartile**  (n=415) | **p-trend^1^** |
| Baseline | 43.00 mL | 45.41 mL | 45.56 mL | 47.87 mL | <0.0001 |
| Year 2 | 49.89 mL | 52.61 mL | 52.28 mL | 55.36 mL | 0.0002 |
| Year 4 | 55.04 mL | 60.24 mL | 57.79 mL | 63.35 mL | 0.0001 |
| ^1^Contrast analysis | | | | | |

| Supplementary Table 6. Adjusted estimate of prostate volume growth in the placebo group (excluding subjects on medicines that might affect glucose/insulin levels) | | | | |
| --- | --- | --- | --- | --- |
| HOMA-IR | **Year 2**  **PV growth relative to baseline** | **95 %CI** | **Year 4**  **PV growth relative to baseline (ml)** | **95% CI** |
| 1^st^ Quartile  2^nd^ Quartile  3^rd^ Quartile  4^th^ Quartile  Linear Contrast | 6.89 mL  6.67 mL  5.90 mL  6.82 mL  P=0.80 | 2.98-10.8 mL  2.81-10.5 mL  2.02-9.78 mL  3.01-10.6 mL | 11.4 mL  13.4 mL  10.4 mL  13.6 mL  P=0.43 | 6.80-16.0 mL  8.83-17.9 mL  5.84-15.0 mL  9.12-18.1 mL |
| Adjusted for baseline data on BMI, PSA, Age, Testosterone, DHT, smoking status, race, region, abnormal DRE and year | | | | |
